# Supplementary material for: VPS13C/PARK23 initiates lipid transfer and membrane remodeling for efficient lysosomal repair
Source: Nat Commun. 2026 Jul 2;17:5789. doi: 10.1038/s41467-026-75145-y (PMC13328759; doi:10.1038/s41467-026-75145-y)
Supplement: Supplementary file 1 — Supplementary Information [file 41467_2026_75145_MOESM1_ESM.pdf]

SUPPLEMENTARY INFORMATION

VPS13C/PARK23 initiates lipid transfer and membrane remodeling for efficient lysosomal repair

Oluwatobi Andrew Adeosun[1,2*], Christian Schröer[1,2*], Elisabeth Südhoff[1,2*], Fabio Bergenthal[1,2*], Katharina Sommer[1,2], Emely Döffinger[1,2], Britta Fiedler[1,2], Angelika Hilderink[1,2], Ann-Katrin Lehmann[1,2], Lea Sophie Pohle[1,2], Sergei M. Korneev[1,2], Florian Fröhlich[2,3], Kenji Maeda[4], Michael Holtmannspötter[2,5], Bianca M. Esch[2,3], Joost C. M. Holthuis[1,2#]

[1]Molecular Cell Biology Section, Department of Biology/Chemistry, Osnabrück University, 49076 Osnabrück, Germany
[2]Center for Cellular Nanoanalytics, Osnabrück University, 49076 Osnabrück, Germany
[3]Bioanalytical Chemistry Section, Department of Biology/Chemistry, Osnabrück University, 49076 Osnabrück, Germany
[4]Cell Death and Metabolism Group, Center for Autophagy, Recycling and Disease, Danish Cancer Society Research Center, DK-2100 Copenhagen, Denmark
[5]Biophysics Section, Department of Biology/Chemistry, Osnabrück University, 49076 Osnabrück, Germany

*These authors contributed equally: Oluwatobi Andrew Adeosun, Christian Schröer, Elisabeth Südhoff, Fabio Bergenthal

[#]Corresponding author: J. C. M. H. (holthuis@uos.de)

This PDF includes:

Supplementary Figures 1 - 13
Supplementary Note 1: Chemical synthesis and NMR spectra of azido-choline
Supplementary Note 2: Image J Macros
Supplementary References

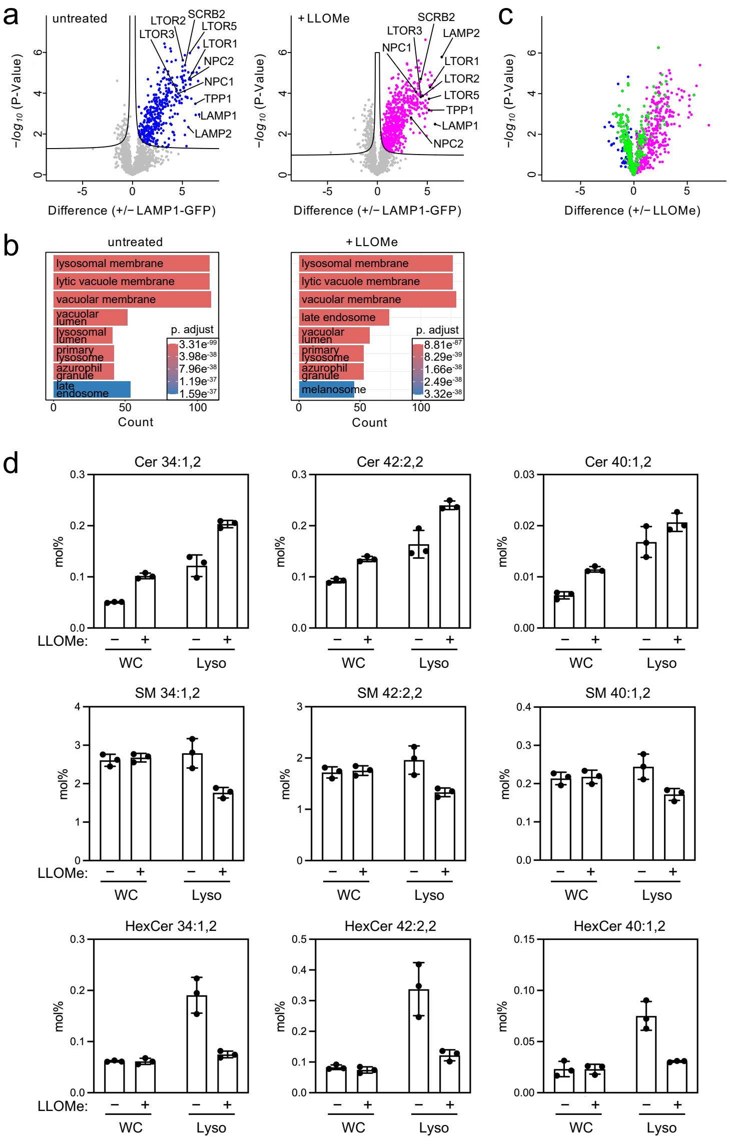

Supplementary Figure 1. Protein and lipid composition of lysosomes affinity-purified from control and LLOMe-treated HeLa cells.

(**a**) Volcano plots showing enrichment of various lysosomal membrane proteins in lysosome isolates from untreated or LLOMe-treated (500 µM, 30 min) LAMP1-GFP-expressing HeLa cells relative to background HeLa cells. Fold changes were calculated from three independent biological replicates and plotted on the x-axis against the negative logarithmic *P*-values on the y-axis.

(**b**) Gene Ontology (GO) enrichment analysis of lysosome isolates relative whole cells. Shown are data from LAMP1-GFP-expressing HeLa cells treated as in (a).

(**c**) Volcano plot of proteins enriched in lysosome isolates from untreated and LLOMe-treated LAMP1-GFP HeLa cells and grouped according to how lysosomal damage affects their relative levels. *Green*, proteins enriched in both intact (–LLOMe) and damaged (+LLOMe) lysosomes; *blue*, proteins selectively depleted in damaged lysosomes; *magenta*, proteins selectively enriched in damaged lysosomes.

(**d**) Lipid composition of whole cell lysates (WC) and lysosomes purified from untreated and LLOMe-treated (500 µM, 30 min) HeLa cells expressing LAMP1-GFP was determined by mass spectrometry-based shotgun lipidomics. Levels of the different lipid species are expressed as mol% of total identified lipids. SM, sphingomyelin; Cer, ceramide; HexCer, hexosylceramide. Data are means ± SD of three biological replicates. Source data are provided as a Source Data file.

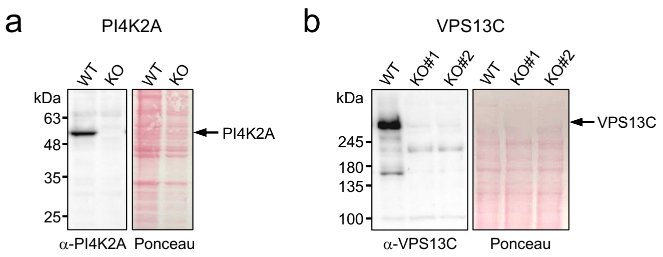

Supplementary Figure 2. Validation of PI4K2A-KO and VPS13C-KO cell lines by immunoblot analysis.

(**a**) U2OS cells lacking PI4K2A were created by CRISPR/Cas9. Loss of PI4K2A was confirmed by immunoblot analysis with an anti-PI4K2A antibody, using Ponceau S staining as loading control. Migration of the PI4K2A protein is marked by an arrow.

(**b**) U2OS cells lacking VPS13C were created by CRISPR/Cas9. Loss of VPS13C was confirmed by immunoblot analysis with an anti-VPS13C antibody, using Ponceau S staining as loading control. Migration of the VPS13C protein is marked by an arrow.

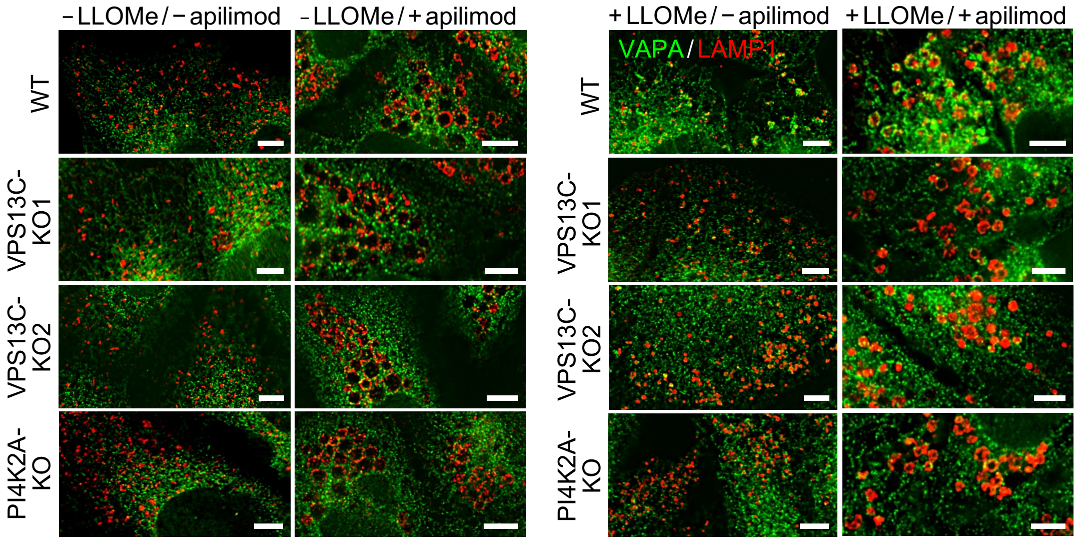

Supplementary Figure 3. VPS13C is essential for tethering damaged lysosomes to the ER.
U2OS wildtype (WT), VPS13C-KO1, VPS13C-KO2 and PI4K2A-KO cells were treated with apilimod (200 nM, 2 h) in combination with LLOMe (1 mM, 10 min), immunostained for LAMP1 (*red*) and VAPA (green), and imaged by DeltaVision microscopy. Scale bar, 5 µm.

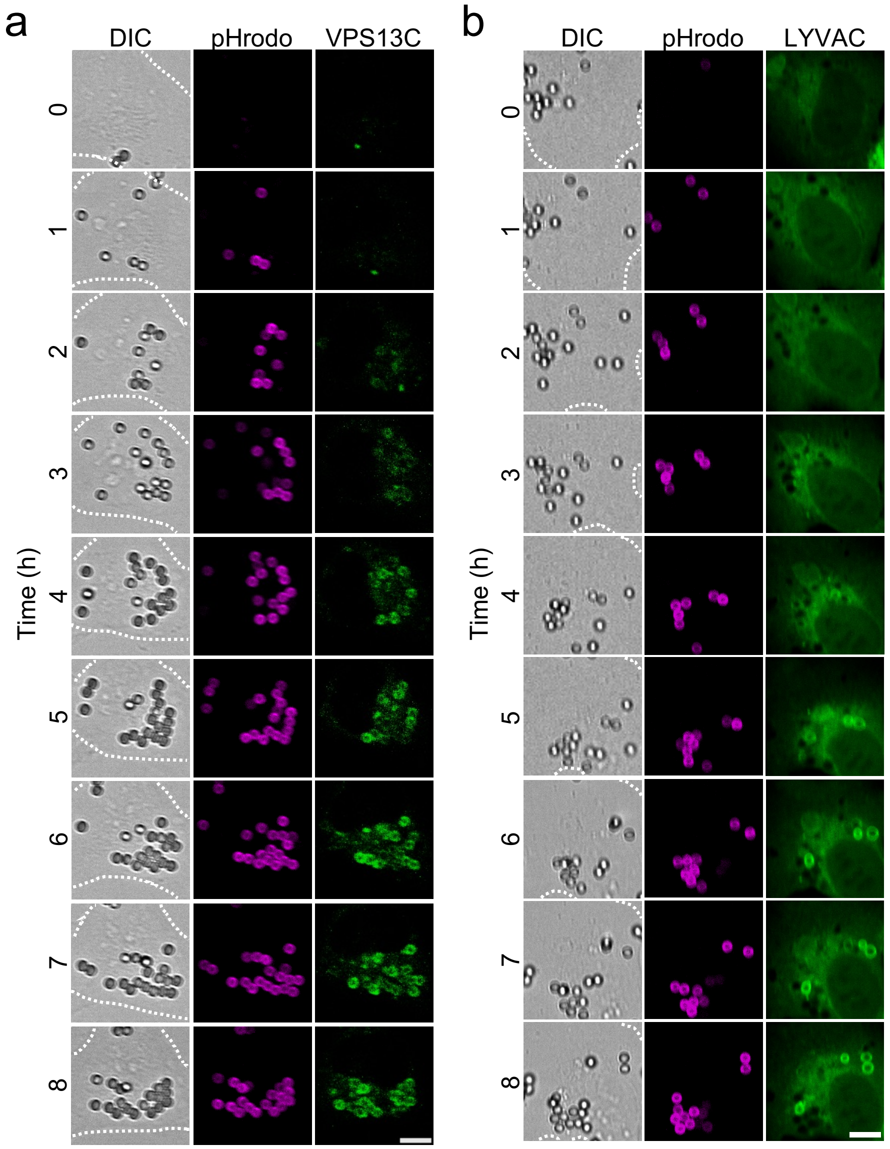

Supplementary Figure 4. pHrodo-conjugated beads internalized by U2OS cells readily gain pHrodo fluorescence and induce large-scale mobilization of VPS13C.

(**a**) Time-lapse images of U2OS cells transfected with VPS13C^mClover (*green*) and incubated with 3 μm pHrodo-microbeads (*magenta*). Cells were imaged by CQ1 spinning disk microscopy. Scale bar, 10 μm.

(**b**) Time-lapse images of U2OS cells transfected with LYVAC-GFP (*green*) and incubated with 3 μm pHrodo-microbeads (*magenta*). Cells were imaged by CQ1 spinning disk microscopy. Scale bar, 10 μm.

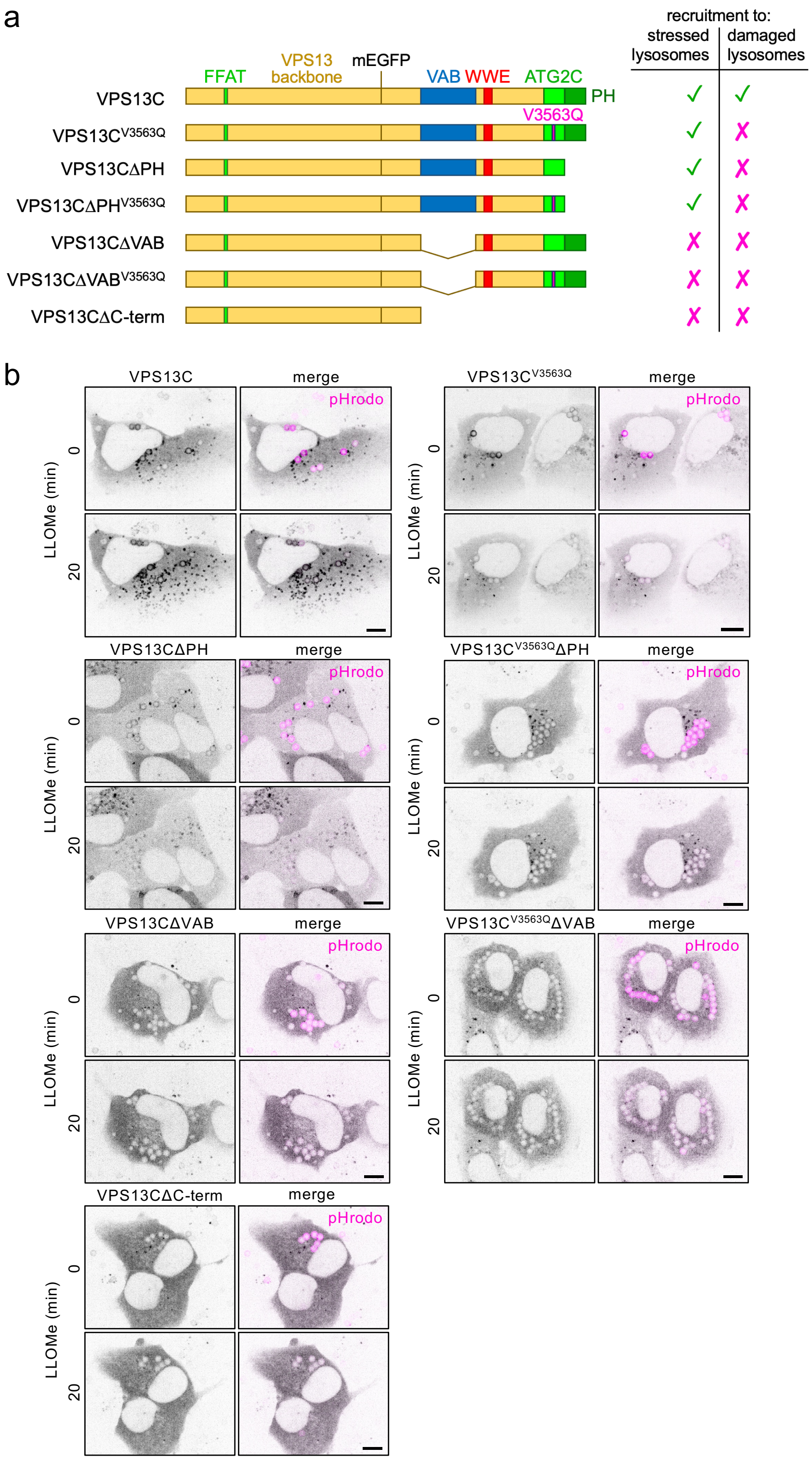

Supplementary Figure 5. The VAB domain of VPS13C is essential for sensing lysosomal stress.
(**a**) VPS13C deletion constructs used for localization studies described in (b).
(**b**) U2OS cells expressing VPS13C^mEGFP deletion constructs as in (a) were fed pHrodo-microbeads (*magenta*) and treated with 1 mM LLOMe for the indicated time. Cells were imaged by spinning disk microscopy. Scale bar, 10 μm.

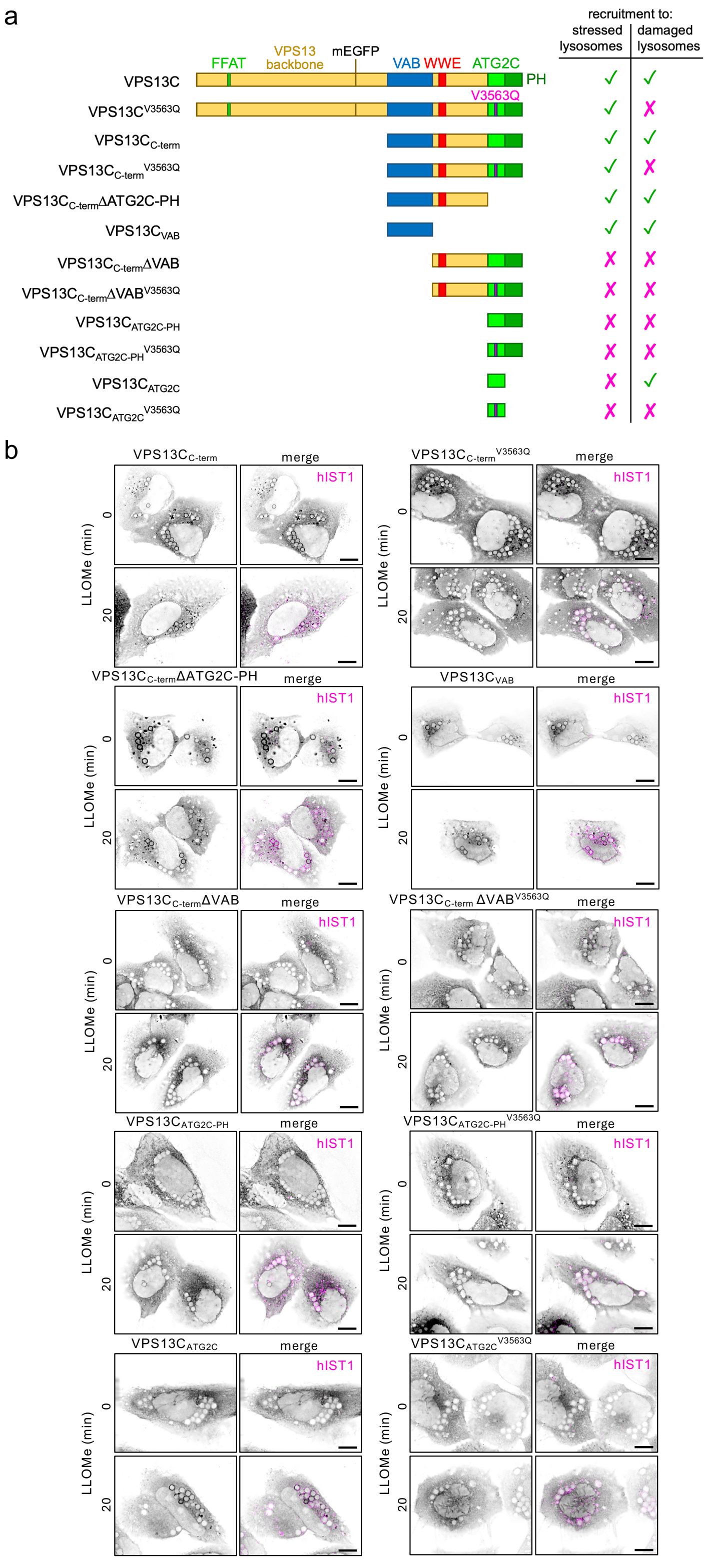

Supplementary Figure 6. VPS13C relies on an intact ATG2C domain for sensing lysosomal damage.
(**a**) VPS13C deletion constructs used for localization studies described in (b).
(**b**) U2OS cells expressing *C*-terminally Halo-tagged VPS13C deletion constructs as in (a) were fed polystyrene microbeads, treated with 1 mM LLOMe for the indicated time, and then immunostained for hIST1 (*magenta*). Cells were imaged by DeltaVision microscopy. Scale bar, 10 µm.

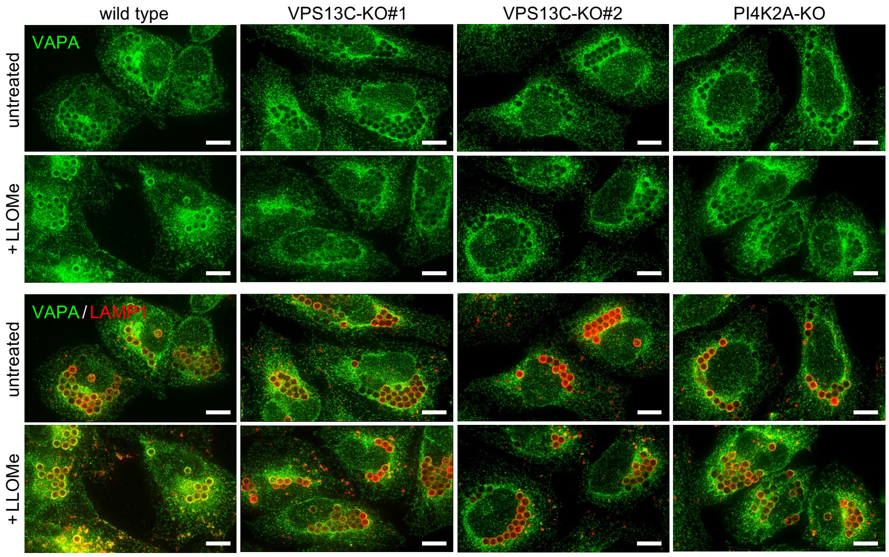

Supplementary Figure 7. VPS13C is required for tethering damaged microbead-containing lysosomes to the ER.
U2OS wildtype (WT), VPS13C-KO1, VPS13C-KO2 and PI4K2A-KO cells were fed 3 μm polystyrene beads, treated with LLOMe (1 mM, 20 min) as indicated, immunostained for LAMP1 (*red*) and VAPA (*green*), and imaged by DeltaVision microscopy. Scale bar, 10 μm.

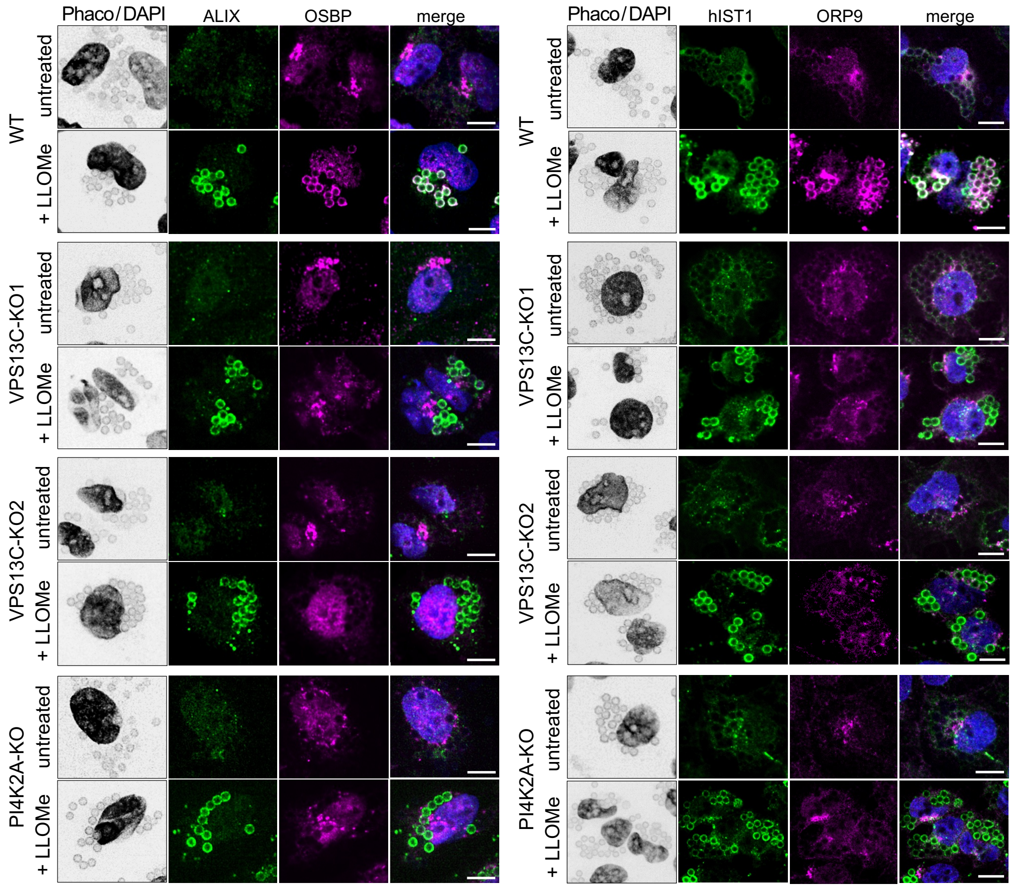

Supplementary Figure 8. VPS13C is essential for recruiting OSBP and ORP9 to damaged lysosomes.

Phase contrast (Phaco) and fluorescence images of polystyrene bead-containing untreated or LLOMe-treated (1 mM, 10 min) U2OS wildtype (WT), VPS13C-KO1, VPS13C-KO2 or PI4K2A-KO cells immunostained for OSBP (*magenta*) and ALIX (*green*) or ORP9 (*magenta*) and hIST1 (*green*) and counterstained with DAPI (*blue*). Cells were imaged by spinning disk microscopy. Scale bar, 10 µm.

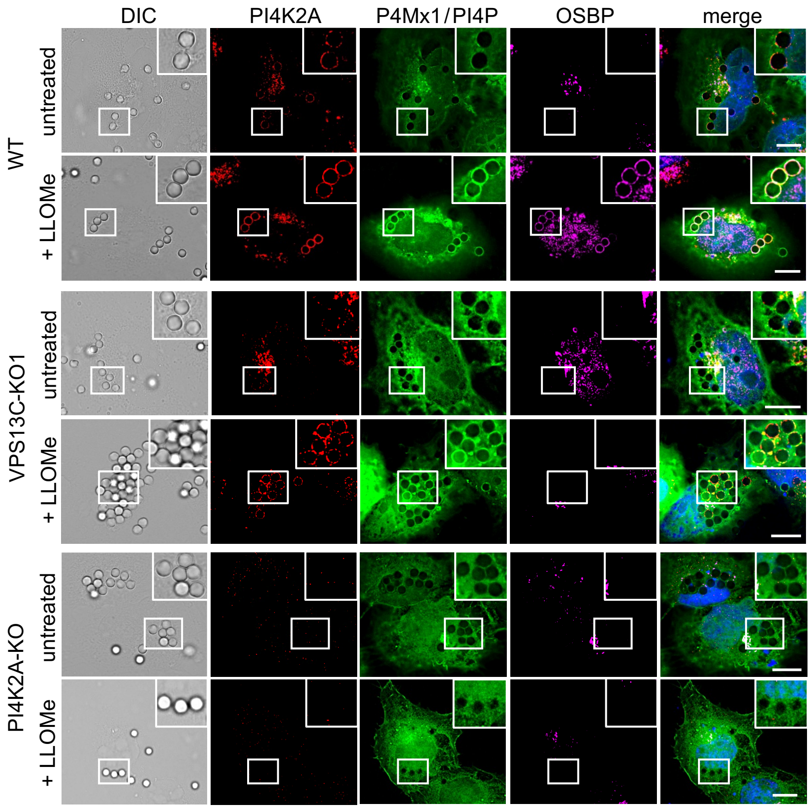

Supplementary Figure 9. Mobilization of PI4K2A to damaged lysosomes is independent VPS13C. Differential interference contrast (DIC) and fluorescence images of polystyrene bead-containing untreated or LLOMe-treated (1 mM, 10 min) U2OS wildtype (WT), VPS13C-KO1 or PI4K2A-KO cells expressing P4MX1-GFP (*green*) and immunostained for PI4K2A (*red*) and OSBP (*magenta*) and counterstained with DAPI (*blue*). Cells were imaged by DeltaVision microscopy. Scale bar, 10 µm.

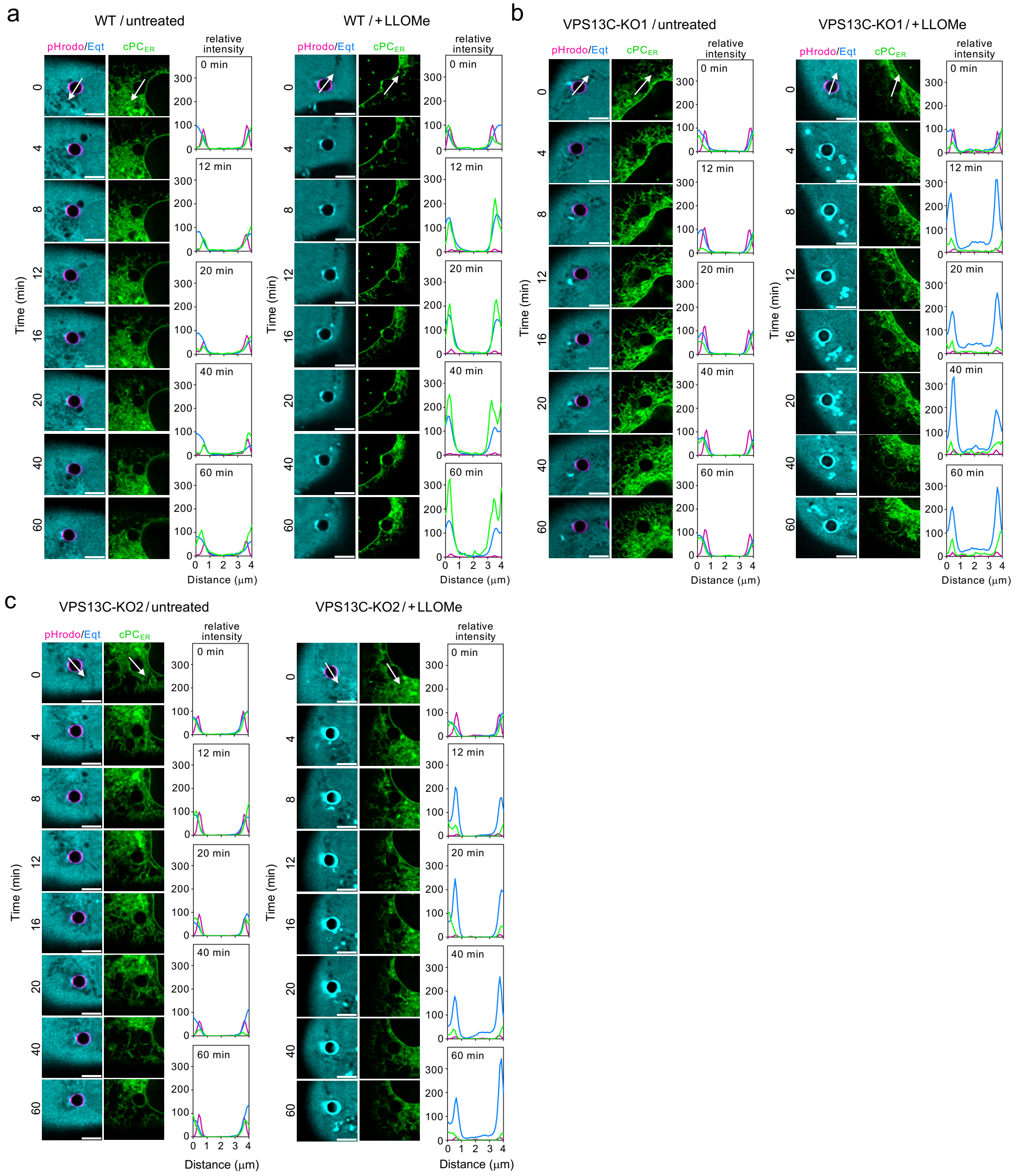

Supplementary Figure 10. VPS13C removal disrupts delivery of cPC$_{ER}$ to damaged lysosomes. (**a, b, c**) U2OS wildtype, VPS13C-KO1 and VPS13C-KO2 cells expressing EqtSM-Halo (*cyan*) were fed pHrodo-microbeads (*magenta*), incubated with N_3-Chol, stained with ER-DBCO (cPC$_{ER}$, *green*) and then subjected to time-lapse imaging in the absence (untreated) or presence of 1 mM LLOMe. Cells were imaged by LLSM and only zoom-ins of imaged cells are shown. Line scans show the intensity profiles of EqtSM-Halo (*cyan*), pHrodo (*magenta*) and cPC$_{ER}$ (*green*) signals along the path of the arrows. Profiles are plotted as relative intensities for each channel normalized to the 0 min time point. Source data are provided as a Source Data file.

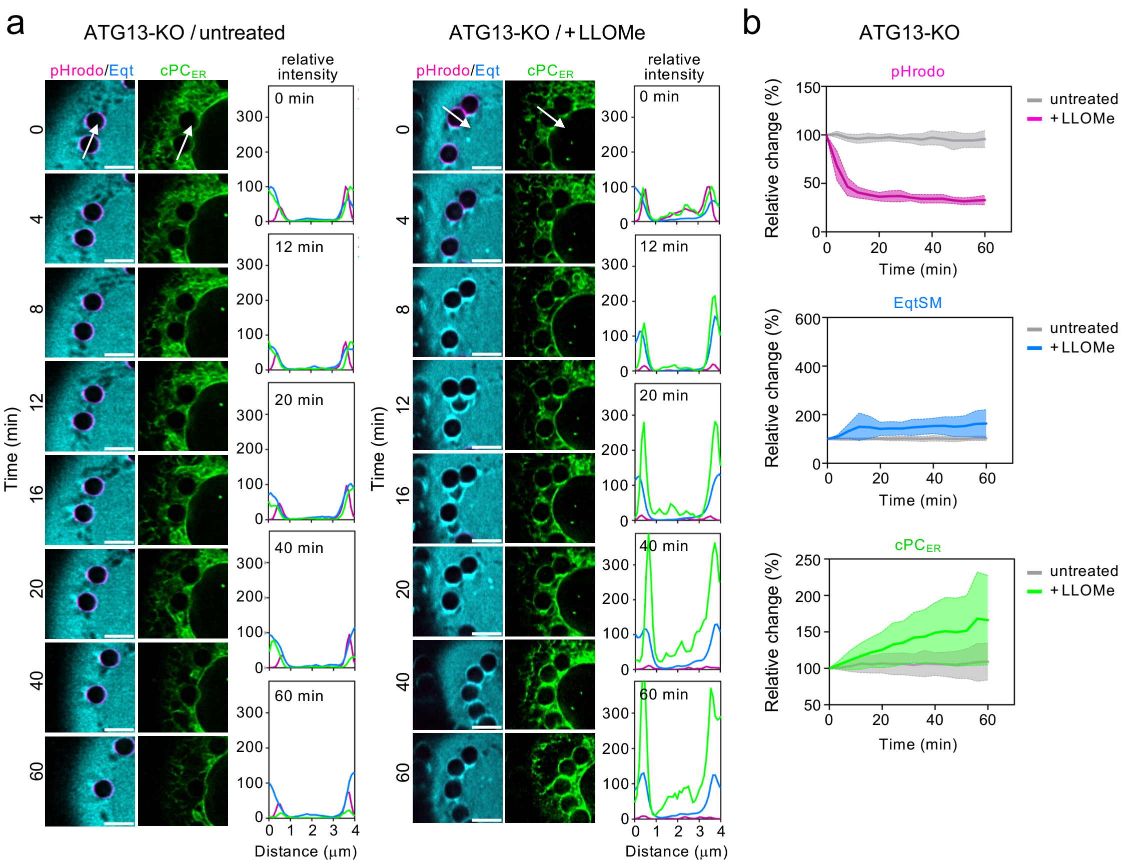

Supplementary Figure 11. Accumulation of cPC$_{ER}$ on damaged lysosomes is not affected in cells defective in autophagy.

(**a**) U2OS ATG13-KO cells expressing EqtSM-Halo (*cyan*) were fed pHrodo-microbeads (*magenta*), incubated with N_3-Chol, stained with ER-DBCO (cPC$_{ER}$, *green*) and then subjected to time-lapse imaging in the absence (untreated) or presence of 1 mM LLOMe. Cells were imaged by LLSM and only zoom-ins of imaged cells are shown. Line scans show the intensity profiles of EqtSM-Halo (*cyan*), pHrodo (*magenta*) and cPC$_{ER}$ (*green*) signals along the path of the arrows. Profiles are plotted as relative intensities for each channel normalized to the 0 min time point. Scale bar, 5 µm.

(**b**) Time course plotting the relative changes in pHrodo, EqtSM-Halo, and cPC$_{ER}$ signals on the surface of pHrodo-microbead containing lysosomes in U2OS ATG13-KO cells treated as in (a). A 3D-surface was generated around the pHrodo-microbeads using Imaris software, and the mean fluorescence intensities of the respective channels on this surface were quantified. For each cell, signals from all bead-associated surfaces were averaged. Values were normalized to the signal at timepoint 0 min and plotted as mean ± SD over time. ATG13-KO (*n* = 3): control, 10 cells; +LLOMe, 9 cells. Source data are provided as a Source Data file.

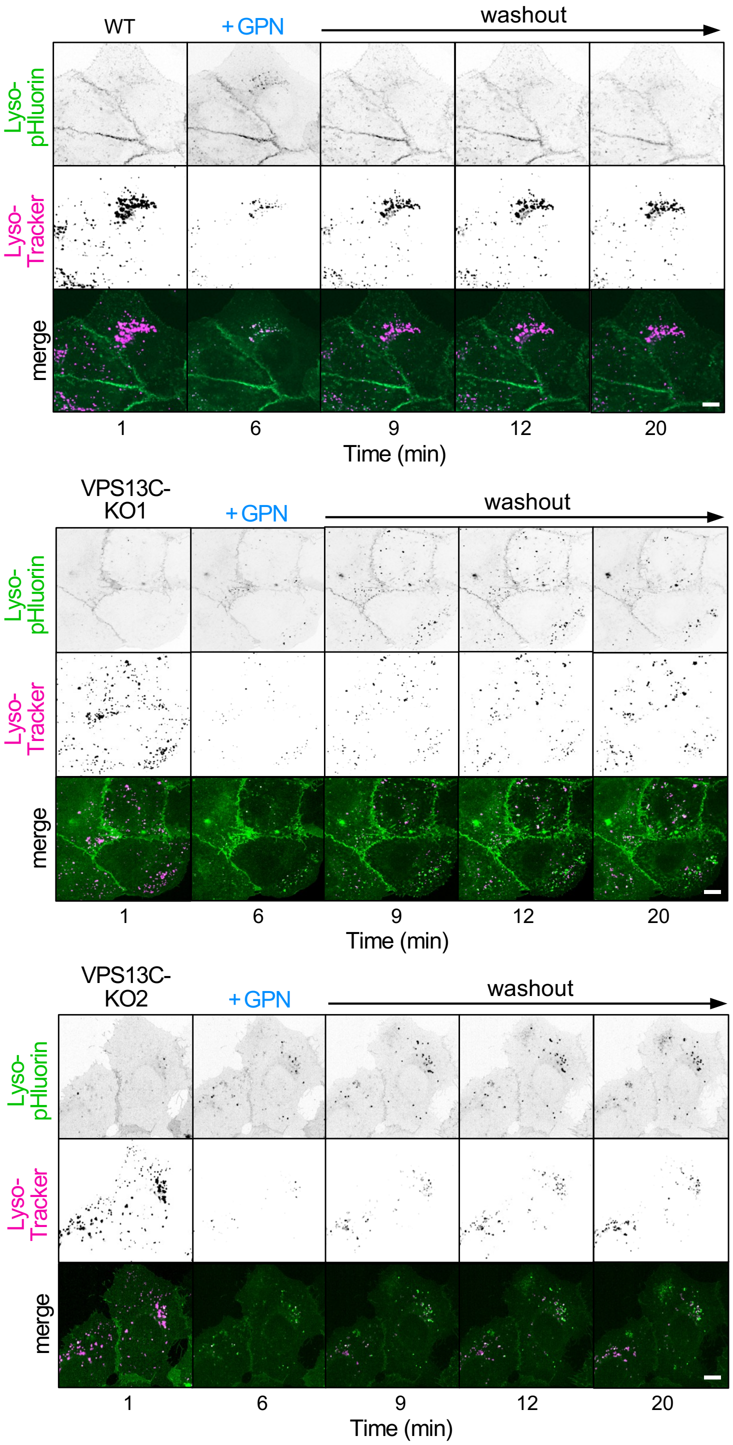

Supplementary Figure 12. VPS13C removal disrupts lysosomal repair.
Time-lapse images of LysoTracker-labelled (*magenta*) and Lyso-pHluorin-expressing (*green*) U2OS wildtype (WT), VPS13C-KO1 and VPS13C-KO2 cells pulse-treated with GPN (200 μM, 2 min). Cells were imaged by spinning disk microscopy. Scale bar, 10 μm.

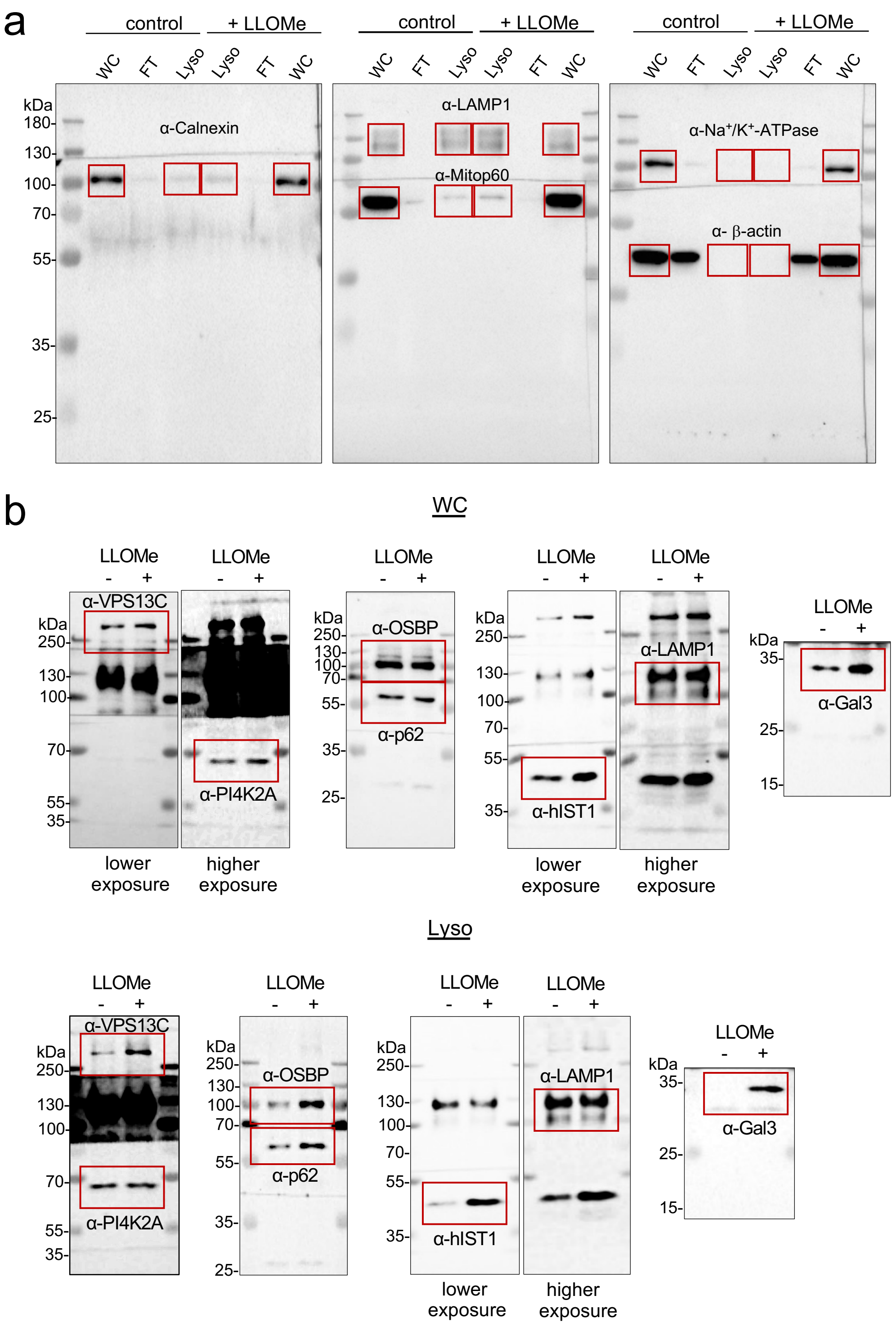

Supplementary Figure 13. Uncropped blots.
(**a**) Uncropped blots of Figure 1c.
(**b**) Uncropped blots of Figure 2b.

Supplementary Note 1

Chemical synthesis of azido-choline

Azido-choline (N_3-Cho) was synthesized in two steps as in Jao *et al.* 2015[1] with some modifications, according to the following scheme:

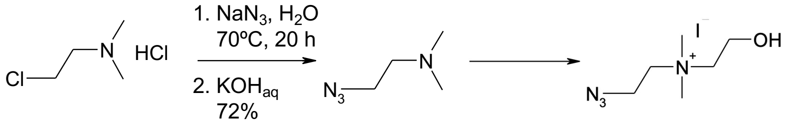

(2-chloroethyl) dimethylamine **ADA** **N$_3$-Chol**

(2-Azidoethyl)dimethylamine (ADA)

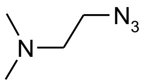

A solution of (2-chloroethyl)dimethylamine hydrochloride (2.88 g, 20 mmol) and sodium azide (3.9 g, 60 mmol) in water (70 ml) was heated at 70°C for 20 h, cooled to room temperature and extracted with diethyl ether (2 x 20 ml). The slightly apricot-colored aqueous phase was basified with aqueous KOH (2 g, 30 mmol) to pH 13 and extracted with diethyl ether (4 x 30 ml), the combined organic extracts were dried (Na_2SO_4), the solvent was carefully distilled off under reduced pressure (550 mm / 27°C) to give azide **ADA** (1.67 g, 73%) as a clear, colourless liquid. The product was found to be of high purity (NMR) and used in the next step without further purification.

^{1}H NMR (250, $CDCl_3$): δ 3.37 (t, *J* = 6.15 Hz, 2H, H_2CN_3), 2.53 (t, *J* = 6.25 Hz, 2H, HCN), 2.30 (s, 6H, 2 x CH_3).

The ^{1}H NMR spectrum is in good agreement with that previously reported[2].

(2-Azidoethyl)-(2-hydroxyethyl)dimethylammonium iodide (N$_3$-Cho)

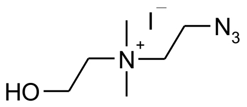

A solution of azide **ADA** (1.14 g, 10 mmol) and iodoethanol (1.81 g, 10.5 mmol) in THF (20 ml) was stirred at 45°C for 30 h and left overnight in a refrigerator (-5°C). The solvent was separated from the white solid mass, the residue was suspended in diethyl ether (10 ml), washed with ether (5 x 10 ml) and dried in vacuo (1 mm, 25°C). The residue was dissolved in MeOH (3 ml) and reprecipitated by adding ether (10 ml), the solution was separated, the residue was washed with ether, dried in vacuo (1 mm, 25°C) and 1.2 g (42 %) of a white N_3-Cho powder was obtained. The product is very hygroscopic.

^{1}H NMR (500, CD_3OD): δ 4.05-4.02 (m, 2H), 4.01-3.98 (m, 2H), 3.72-3.69 (m, 2H), 3.61-3.59 (m, 2H), 3.26 (s, 6H, 2 x CH_3).

^{13}C NMR (500, CD_3OD): δ 66.28, 63.36, 55.48, 51.63 (CH_3), 44.73.

The ^{1}H and ^{13}C NMR spectra are in good agreement with those previously reported[1].

NMR spectra

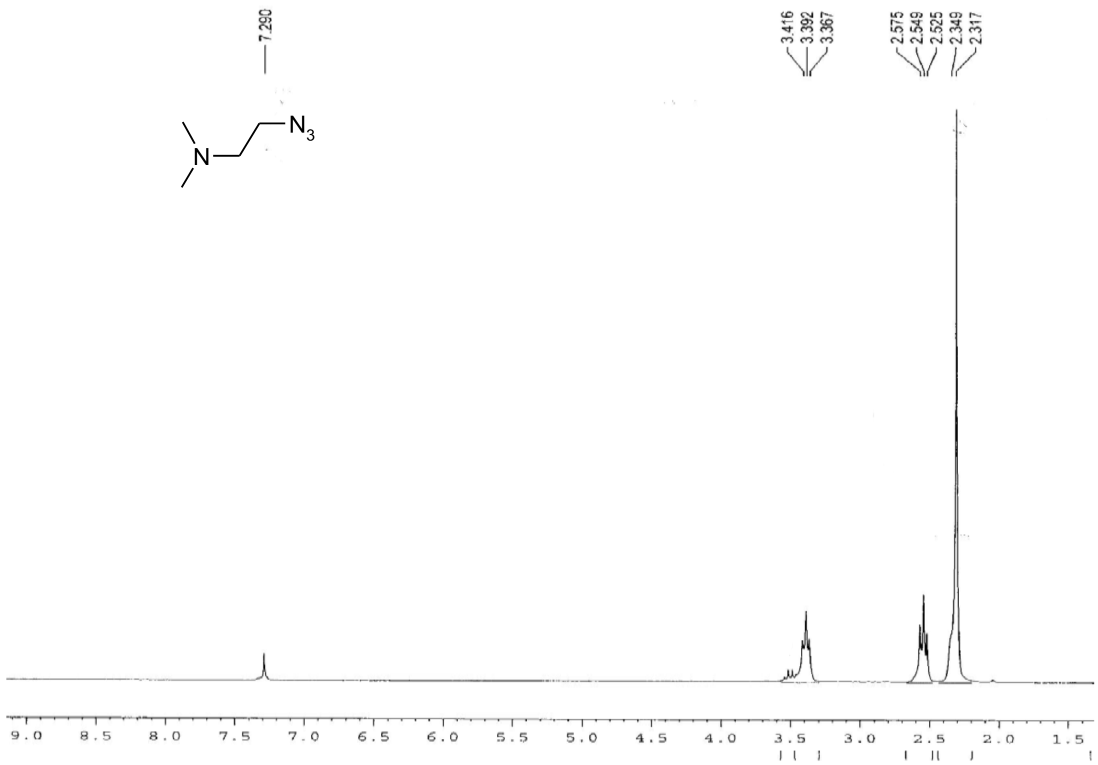

^{1}H NMR (250 MHz, $CDCl_3$) of (2-azidoethyl)dimethylamine (ADA)

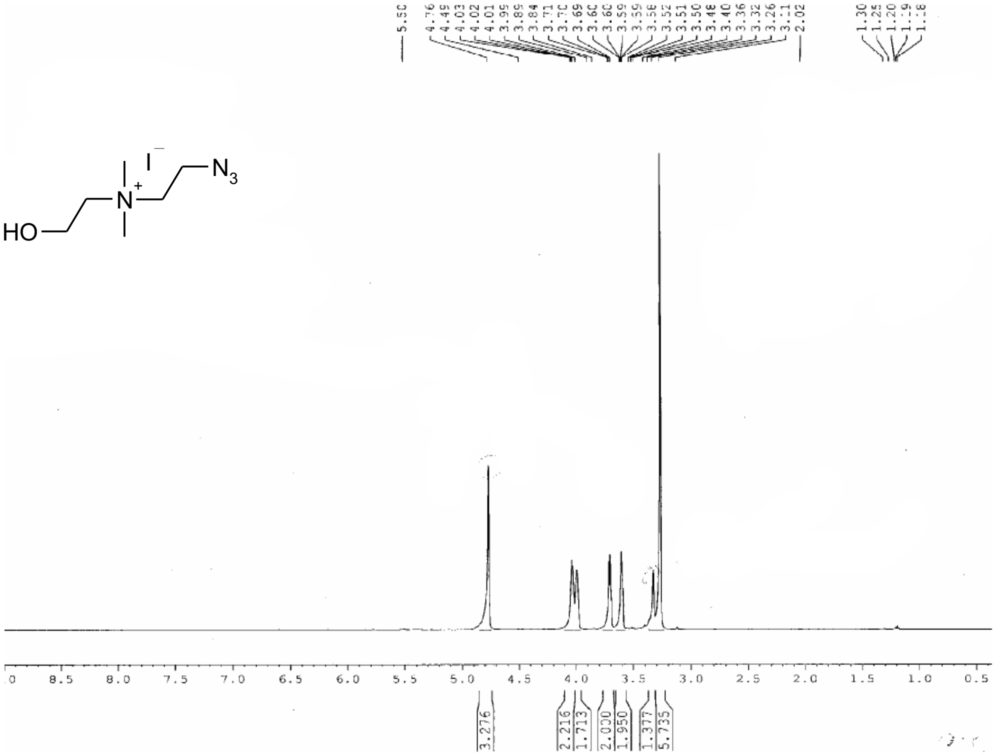

^{1}H NMR (500 MHz, CD_3OD) of azido-choline (N_3-Chol). The signal at 4.76 ppm originates from residual H_2O in CD_3OD (3.36 ppm).

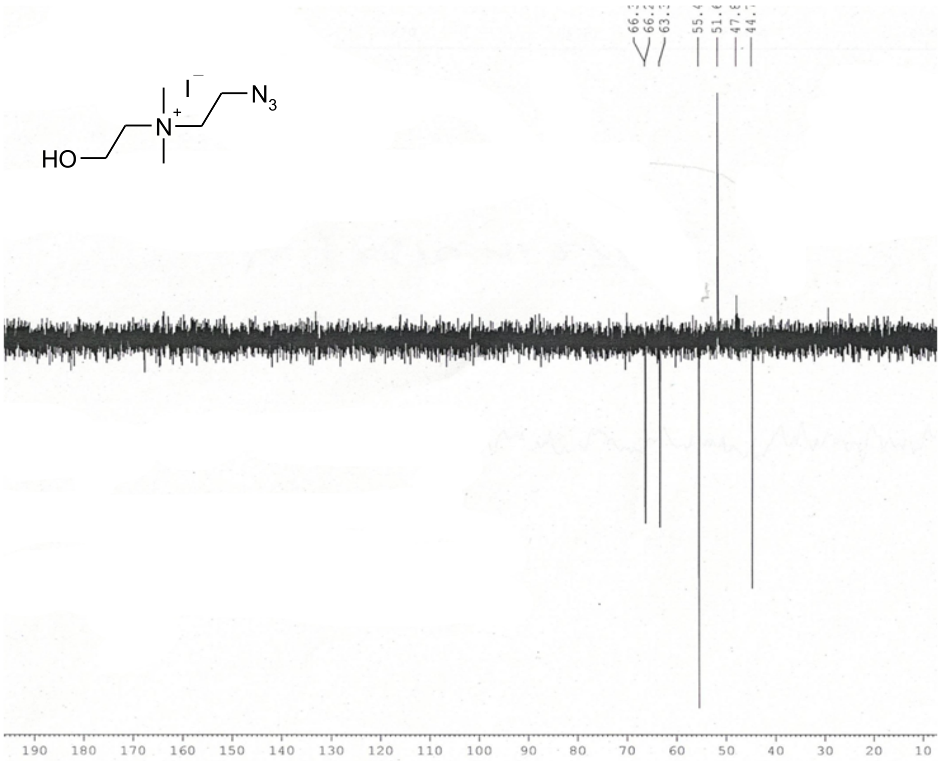

^{13}C NMR DEPT135 (126 MHz, CD_3OD) of azido-choline (N_3-Chol).

Supplementary Note 2

Image J Macros

Quantification of VPS13C^mClover, GFP-OSBP, and LysopHluorin puncta

1. //set Threshold manually
2. setOption("BlackBackground", false);
3. run("Convert to Mask", "method=Default background=Dark");
4. run("Fill Holes","stack");
5. run("Watershed", "stack");
6. run("Analyze Particles...", "size=0.1-5 circularity=0.50-1.00 show=Outlines display exclude summarize stack");
7. run("Next Slice [>]")
8. //Repeat step 6 and 7 until all time points are analyzed

Quantification of VPS13C^mEGFP, VPS13C^{V2563Q}^mEGFP and PDZD8-GFP puncta

1. //set Threshold manually
2. setOption("BlackBackground", false);
3. run("Convert to Mask", "method=Default background=Dark");
4. run("Fill Holes","stack");
5. run("Watershed", "stack");
6. run("Analyze Particles...", "size=0.1-5 circularity=0.2-1.00 show=Outlines display exclude summarize stack");

Quantification of LysoTracker puncta

1. run("Subtract...", "value=20 stack");
2. setAutoThreshold("Default dark no-reset");
3. setOption("BlackBackground", false);
4. run("Convert to Mask", "method=Default background=Dark");
5. run("Convert to Mask", "method=Default background=Light");
6. run("Watershed", "stack");
7. run("Analyze Particles...", "size=0.2-5 circularity=0.5-1.00 show=Outlines display exclude summarize stack");
8. run("Next Slice [>]")
9. // repeat step 7 and 8 until all time frames have been quantified

Quantification of pHrodo, cPC_{ER} and CNX-Halo and EqtSM-Halo signals on pHrodo-microbead containing lysosomes

Macro based on Maib, H. and Murray, D.H. (2022) ‘A mechanism for exocyst-mediated tethering via Arf6 and PIP5K1C-driven phosphoinositide conversion’, *Current Biology*, 32(13), pp. 2821-2833.e6 (https://doi.org/10.1016/j.cub.2022.04.089) with modifications by Steffen Wolke-Hanenkamp (Ultraphysics Division, Osnabrück University).

```
// Check for the Excel plugin
if (File.exists(getDirectory("plugins") + "/Read_and_Write_Excel-1.1.7.jar") != 1) {
    exit("ResultsToExcel Plugin not found. Make sure you activated the plugin in the ImageJ
updater. ('Help' > 'Update...' > 'Manage update sites')");
}
// Get input image and basic parameters
dir1 = getDirectory("image");
name = getTitle();
selectWindow(name);
getDimensions(width, height, channels, slices, frames);
run("Split Channels");
// Open results Excel sheet
run("Read and Write Excel", "file_mode=read_and_open file=[" + dir1 +
"/CS_BeadsResults.xlsx] sheet=[C1]");
// Iterate over all time frames
for (i = 1; i <= frames; i++) {
    // --- MASK GENERATION (Channel 2) ---
    selectWindow("C2-" + name);
    Stack.setFrame(i);
    run("Duplicate...", "title=C2-mask-frame_" + i + ".tif frames=" + i);
    maskID = getImageID();
    setAutoThreshold("Li dark");
    run("Convert to Mask", "method=Li background=Dark calculate");
    setOption("BlackBackground", false);
    run("Fill Holes");
    run("Watershed");
    run("Analyze Particles...", "size=5-250 circularity=0.1-1.00 show=Overlay clear add stack");
    // --- ROI ENLARGEMENT ---
    upperROI = roiManager("count");
    for (index = 0; index < upperROI; index++) {
        roiManager("Select", index);
        run("Enlarge...", "enlarge=-1");
        run("Make Band...", "band=1.25");
        roiManager("Update");
    }
    // --- MEASURE CHANNEL 1 ---
    selectWindow("C1-" + name);
    Stack.setFrame(i);
    run("Duplicate...", "title=C1-double.tif frames=" + i);
    C1doubleID = getImageID();
    roiManager("Deselect");
    roiManager("Measure");
    run("Read and Write Excel", "file_mode=queue_write dataset_label=[Frame " + i + "]
```

```
sheet=[C1]");
   run("Clear Results");
   close(C1doubleID);
   // --- MEASURE CHANNEL 2 ---
   selectWindow("C2-" + name);
   Stack.setFrame(i);
   run("Duplicate...", "title=C2-double.tif frames=" + i);
   C2doubleID = getImageID();
   roiManager("Deselect");
   roiManager("Measure");
   run("Read and Write Excel", "file_mode=queue_write dataset_label=[Frame " + i + "]
sheet=[C2]");
   run("Clear Results");
   close(C2doubleID);
   // --- MEASURE CHANNEL 3 ---
   selectWindow("C3-" + name);
   Stack.setFrame(i);
   run("Duplicate...", "title=C3-double.tif frames=" + i);
   C3doubleID = getImageID();
   roiManager("Deselect");
   roiManager("Measure");
   run("Read and Write Excel", "file_mode=queue_write dataset_label=[Frame " + i + "]
sheet=[C3]");
   run("Clear Results");
   close(C3doubleID);
   // --- SAVE MASK ---
   selectImage(maskID);
   saveAs("PNG", dir1 + File.separator + i + "C2-Masks");
   close(maskID);
}
// Finalize Excel export and clean up
run("Read and Write Excel", "file_mode=write_and_close");
close("*");
close("Results");
close("ROI Manager");
// Notify user
waitForUser("End", "Macro has finished");
```

Supplementary References

1. Jao, C. Y., Roth, M., Welti, R. & Salic, A. Biosynthetic labeling and two-color imaging of phospholipids in cells. *ChemBioChem* **16**, (2015), 472–476. DOI: 10.1002/cbic.201402149.
2. Bernhard, Y., Gigot, E., Goncalves, V., Moreau, M., Sok, N., Richard, P. & R. A. Decréau. Direct subphthalocyanine conjugation to bombesin vs. indirect conjugation to its lipidic nanosarrier. *Org. Biomol. Chem*. **14**, (2016). 4511–4518. DOI: 10.1039/C6OB00530F.
