# Supplementary material for: VPS13C/PARK23 initiates lipid transfer and membrane remodeling for efficient lysosomal repair
Source: Nat Commun. 2026 Jul 2;17:5789. doi: 10.1038/s41467-026-75145-y (PMC13328759; doi:10.1038/s41467-026-75145-y)
Supplement: Supplementary file 2 — Description of Additional Supplementary Files [file 41467_2026_75145_MOESM2_ESM.pdf]

Description of Additional Supplementary Files

File name: Supplementary Data 1
Description: List of primers used for cloning and site-directed mutagenesis.

File name: Supplementary Data 2
Description: List of internal lipid standards used for shotgun lipidomics.

File name: Supplementary Data 3
Description: List of class-specific criteria used for lipid identifications.
