# Supplementary material for: VPS13C/PARK23 initiates lipid transfer and membrane remodeling for efficient lysosomal repair
Source: Nat Commun. 2026 Jul 2;17:5789. doi: 10.1038/s41467-026-75145-y (PMC13328759; doi:10.1038/s41467-026-75145-y)
Supplement: Supplementary file 6 — Reporting Summary [file 41467_2026_75145_MOESM6_ESM.pdf]

nature portfolio

Corresponding author(s): Joost Holthuis

Last updated by author(s): Jun 9, 2026

Reporting Summary

Nature Portfolio wishes to improve the reproducibility of the work that we publish. This form provides structure for consistency and transparency in reporting. For further information on Nature Portfolio policies, see our Editorial Policies and the Editorial Policy Checklist.

Statistics

For all statistical analyses, confirm that the following items are present in the figure legend, table legend, main text, or Methods section.

n/a	Confirmed	
☐	☒	The exact sample size (*n*) for each experimental group/condition, given as a discrete number and unit of measurement
☐	☒	A statement on whether measurements were taken from distinct samples or whether the same sample was measured repeatedly
☐	☒	The statistical test(s) used AND whether they are one- or two-sided<br>*Only common tests should be described solely by name; describe more complex techniques in the Methods section.*
☒	☐	A description of all covariates tested
☐	☒	A description of any assumptions or corrections, such as tests of normality and adjustment for multiple comparisons
☐	☒	A full description of the statistical parameters including central tendency (e.g. means) or other basic estimates (e.g. regression coefficient) AND variation (e.g. standard deviation) or associated estimates of uncertainty (e.g. confidence intervals)
☐	☒	For null hypothesis testing, the test statistic (e.g. *F*, *t*, *r*) with confidence intervals, effect sizes, degrees of freedom and *P* value noted<br>*Give P values as exact values whenever suitable.*
☒	☐	For Bayesian analysis, information on the choice of priors and Markov chain Monte Carlo settings
☒	☐	For hierarchical and complex designs, identification of the appropriate level for tests and full reporting of outcomes
☒	☐	Estimates of effect sizes (e.g. Cohen's *d*, Pearson's *r*), indicating how they were calculated

Our web collection on statistics for biologists contains articles on many of the points above.

Software and code

Policy information about availability of computer code

Data collection

Immunofluorescence images were captured using an Olympus IX-71 DeltaVision Elite microscope equipped with a pco.edge 4.2 (PCO) camera and an Olympus PLAPON 60x (NA 1.42) oil immersion objective. Time-lapse recoding of cellular uptake of pHrodo-microbeads was performed using a Yokogawa CQ1 Confocal Spinning Disk Microscope with temperature, CO2, O2 and humidity control, a microlense-enhanced dual Nipkow disk system, a camera with a sCMOS chip, a laser-based hardware autofocus and an Olympus IPLSAPO40X2 40x (NA 0.95) air objective. Time-lapse recording of cells exposed to lysosome-damaging drugs was performed using a Zeiss Cell Observer Spinning Disc Confocal Microscope equipped with a TempModule S1 temperature control unit, a Yokogawa Spinning Disc CSU-X1a 5000 Unit, an Evolve EMCCDD camera (Photonics, Tucson), a motorized xyz-stage PZ-2000 XYZ (Applied Scientific Instrumentation) and an Alpha Plan-Apochromat x63 (NA 1.46) oil immersion objective. Lyso Flipper-TR fluorescence lifetime imaging (FLIM) was performed using an Olympus IX-81 confocal laser scanning microscope (FV-1000) equipped with a compact lifetime and FCS upgrade kit for LSMs from PicoQuant using a 60x oil immersion objective (UPLXAPO 60x, NA 1.42; Olympus). Lyso Flipper-TR was excited using a pulsed 485 nm laser (LDH-D-C-485; PicoQuant) at a pulse rate of 20 MHz. Emitted photons were filtered with a dichroic mirror (HC-BS560; AHF Analysentechnik) and a bandpass filter (BrightLine HC 607/37; AHF Analysentechnik) and collected with a Hybrid Photomultiplier Detector (PMA Hybrid-40; PicoQuant). Lattice light-sheet microscopy (LLSM) was performed on a home-built clone of the original design by the Betzig group (PMID: 25342811). Protein mass spectrometry was performed using a Thermo Ultimate 3000 RSLCnano system connected to a TimsTOF HT mass spectrometer (Bruker Corporation, Bremen) through a captive spray ion source. Peptides were separated on an Aurora Gen3 C18 column (25 cm x 75 µm x 1.6 µm) with CSI emitter (Ionoptics, Australia) at 40°C. Eluted peptides were electrosprayed into the mass spectrometer at an electrospray voltage of 1.5 kV and 3 l/min dry gas. MS settings were adjusted to positive ion polarity and an MS range from 100 to 1700 m/z. The scan mode was set to DIA-PASEF. Lipid mass spectrometry was performed in the negative and positive ionization modes using a Q Exactive Hybrid Quadrupole-Orbitrap mass spectrometer (Thermo Fisher Scientific, Waltham, MA) coupled to TriVersa NanoMate (Advion Biosciences, Ithaca, NY, USA).

Data analysis

Fluorescence images captured with the Olympus IX-71 DeltaVision Elite microscope were deconvoluted using SoftWoRx software and further

Data analysis	processed with Fiji Image J2 software (version 2.3.0/1.53f; National Institute of Health, USA). Fluorescence images captured with the Zeiss Cell Observer Spinning Disc Confocal Microscope were acquired using Zeiss Zen 2012 acquisition software. FLIM images captured with the Olympus IX-81 confocal laser scanning microscope were analyzed using SymPhoTime64 (PicoQuant). Fluorescence images captured with the lattice light-sheet microscope were processed using an open-source LLSM post-processing utility called LLSpy (https://github.com/tlambert03/LLSpy) for deskewing, deconvolution, channel registration and transformation. Analysis of microscopy images was performed using Image J macros on the original, unmodified data. Macros are included in the Online Methods section of the manuscript. Protein mass spectrometry data were analysed using MaxQuant (V2.7.0.0 www.maxquant.org) and Perseus (V2.1.5.0 www.maxquant.org/perseus). Immunoblot band intensities were quantified using Image Lab 6.0.1 software (Bio-Rad Laboratories). Volcano plots were generated with the R software package (www.rproject.org/; RRID:SCR_001905). The lipidomics data were processed using LipidXplorer (version 1.2.4) and lipid species were identified using class-specific criteria, provided as Supplementary Data 3.

For manuscripts utilizing custom algorithms or software that are central to the research but not yet described in published literature, software must be made available to editors and reviewers. We strongly encourage code deposition in a community repository (e.g. GitHub). See the Nature Portfolio guidelines for submitting code & software for further information.

Data

Policy information about availability of data

All manuscripts must include a data availability statement. This statement should provide the following information, where applicable:

- Accession codes, unique identifiers, or web links for publicly available datasets
- A description of any restrictions on data availability
- For clinical datasets or third party data, please ensure that the statement adheres to our policy

All data generated or analysed during this study are included in the manuscript and supporting files. Source Data are provided for Figures 1-6, 8-10 and Supplemental Figures 1, 9 and 10. Uncropped blots of Figures 1c and 2b are provided in Supplementary Figure 13. The mass spectrometry proteomics data have been deposited to the ProteomeXchange Consortium via the PRIDE partner repository65 with the dataset identifier PXD069825 (https://www.ebi.ac.uk/pride/archive/projects/PXD069825/). The shotgun lipidomics data have been deposited to MetaboLights with the dataset identifier MTBLS14737 (https://www.ebi.ac.uk/metabolights/MTBLS14737).

Research involving human participants, their data, or biological material

Policy information about studies with human participants or human data. See also policy information about sex, gender (identity/presentation), and sexual orientation and race, ethnicity and racism.

Reporting on sex and gender	*Use the terms sex (biological attribute) and gender (shaped by social and cultural circumstances) carefully in order to avoid confusing both terms. Indicate if findings apply to only one sex or gender; describe whether sex and gender were considered in study design; whether sex and/or gender was determined based on self-reporting or assigned and methods used. Provide in the source data disaggregated sex and gender data, where this information has been collected, and if consent has been obtained for sharing of individual-level data; provide overall numbers in this Reporting Summary. Please state if this information has not been collected. Report sex- and gender-based analyses where performed, justify reasons for lack of sex- and gender-based analysis.*
Reporting on race, ethnicity, or other socially relevant groupings	*Please specify the socially constructed or socially relevant categorization variable(s) used in your manuscript and explain why they were used. Please note that such variables should not be used as proxies for other socially constructed/relevant variables (for example, race or ethnicity should not be used as a proxy for socioeconomic status). Provide clear definitions of the relevant terms used, how they were provided (by the participants/respondents, the researchers, or third parties), and the method(s) used to classify people into the different categories (e.g. self-report, census or administrative data, social media data, etc.) Please provide details about how you controlled for confounding variables in your analyses.*
Population characteristics	*Describe the covariate-relevant population characteristics of the human research participants (e.g. age, genotypic information, past and current diagnosis and treatment categories). If you filled out the behavioural & social sciences study design questions and have nothing to add here, write "See above."*
Recruitment	*Describe how participants were recruited. Outline any potential self-selection bias or other biases that may be present and how these are likely to impact results.*
Ethics oversight	*Identify the organization(s) that approved the study protocol.*

Note that full information on the approval of the study protocol must also be provided in the manuscript.

Field-specific reporting

Please select the one below that is the best fit for your research. If you are not sure, read the appropriate sections before making your selection.

☒ Life sciences ☐ Behavioural & social sciences ☐ Ecological, evolutionary & environmental sciences

For a reference copy of the document with all sections, see nature.com/documents/nr-reporting-summary-flat.pdf

Life sciences study design

All studies must disclose on these points even when the disclosure is negative.

Sample size	Sample sizes were determined based on the author's experience of what is necessary to generate a convincing and compelling result.
Data exclusions	As documented in the source data file, we excluded 2 of the total 288 data points in Figure 6h from the analysis because they were clearly outliers. We did not pre-establish any exclusion criteria.
Replication	Each experiment was repeated at least twice with similar results using independent experimental samples and statistical tests as specified in the figure legends. Source data with sample sizes, number of technical and/or biological replicates, means, standard deviations, and calculated p values (where applicable) are provided in the Source Data file.
Randomization	N/A
Blinding	No blinding was done in this study. Virtually all the data are quantitative. Most measurements were made using a machine and not easily subject to operator bias.

Reporting for specific materials, systems and methods

We require information from authors about some types of materials, experimental systems and methods used in many studies. Here, indicate whether each material, system or method listed is relevant to your study. If you are not sure if a list item applies to your research, read the appropriate section before selecting a response.

Materials & experimental systems

n/a	Involved in the study	
☐	☒	Antibodies
☐	☒	Eukaryotic cell lines
☒	☐	Palaeontology and archaeology
☒	☐	Animals and other organisms
☒	☐	Clinical data
☒	☐	Dual use research of concern
☒	☐	Plants

Methods

n/a	Involved in the study	
☒	☐	ChIP-seq
☒	☐	Flow cytometry
☒	☐	MRI-based neuroimaging

Antibodies

Antibodies used	Antibodies used were: rabbit polyclonal anti-hIST1 (Proteintech, 19842; IB: 1:1000; IF 1:1000), rabbit polyclonal anti-OSBP (Proteintech, 11096-1-AP; IB: 1:500; IF: 1:200), mouse monoclonal anti-PI4-kinase II Alpha (B5) (Santa Cruz, sc-390026; IB 1:1000; IF 1:400), rabbit polyclonal anti-VPS13C (Proteintech, 28676-1-AP; IB 1:1000), mouse monoclonal anti-α-Actin (Sigma, A1978; IB 1:50,000), mouse monoclonal anti-Mitochondrial surface p60 (Millipore, MAB1273; IB 1:1000), mouse monoclonal anti-Na+/K+ ATPase (Santa Cruz, sc-48345; IB 1:1000), mouse monoclonal anti-LAMP1 (H4A3) (Santa Cruz, sc-20011; IB 1:1000; IF 1:400), rabbit polyclonal anti-Calnexin (Santa Cruz, sc-11397; IB 1:1000), mouse monoclonal anti-Galectin 3 (B2C10) (Santa Cruz, sc-32790; IB 1:1000), mouse monoclonal anti-VAPA (4C12) (Santa Cruz, sc-293278; IF 1:400), mouse monoclonal anti-p62/SQSTM (D-3) (Santa Cruz, sc-28359; IB 1:500), mouse monoclonal anti-Alix (BioLegend, 634502; IF 1:200), mouse monoclonal anti-ORP-9 (A7) (Santa Cruz, sc-398961; IF 1:200), HRP-conjugated goat anti-mouse IgG (Thermo Fisher Scientific, 31430; IB 1:5000), HRP-conjugated goat anti-rabbit IgG (Thermo Fisher Scientific; 31460; IB 1:5000), Cyanine Cy™2-conjugated donkey anti-mouse IgG (Jackson ImmunoResearch Laboratories, 715-225-150; IF 1:250), Cyanine Cy™2-conjugated donkey anti-rabbit IgG (Jackson ImmunoResearch Laboratories, 715-225-152; IF 1:250), Cyanine Cy™3-conjugated donkey anti-mouse IgG (Jackson ImmunoResearch Laboratories, 715-162-150; IF 1:250), Cyanine Cy™3-conjugated donkey anti-rabbit IgG (Jackson ImmunoResearch Laboratories, 711-165-152; IF 1:250), Cyanine Cy™5-conjugated donkey anti-mouse IgG (Jackson ImmunoResearch Laboratories, 715-175-150; IF 1:250), and Cyanine Cy™5-conjugated donkey anti-rabbit IgG (Jackson ImmunoResearch Laboratories, 711-175-152; IF 1:250).
Validation	Specificity of anti-VPS13C and anti-PI4K2A antibodies was verified by immunoblot analysis of total lysates from VPS13C-KO and PI4K2A-KO U2OS cells. All other commercial antibodies were validated by the suppliers.

Eukaryotic cell lines

Policy information about cell lines and Sex and Gender in Research

Cell line source(s)	Human cervical carcinoma HeLa (ATCC CCL-2), human osteosarcoma U2OS (ATCC HTB-96), human embryonic kidney 293 cells transformed with Simian Virus 40 large T antigen (HEK293T, ATCC CRL-3216). U2OS ATG13-KO cells were provided by Fulvio Reggiori (University of Aarhus, Denmark) and described in Mauthe et al. (Ref. 55).
Authentication	Cell line authentication was not performed as cells were not listed in the commonly misidentified category.

Mycoplasma contamination	Cell-lines were routinely screened for mycoplasma infection by PCR test. All experiments were performed using cells free of mycoplasma.
Commonly misidentified lines (See ICLAC register)	No commonly misidentified cell lines were used.

Plants

Seed stocks	*Report on the source of all seed stocks or other plant material used. If applicable, state the seed stock centre and catalogue number. If plant specimens were collected from the field, describe the collection location, date and sampling procedures.*
Novel plant genotypes	*Describe the methods by which all novel plant genotypes were produced. This includes those generated by transgenic approaches, gene editing, chemical/radiation-based mutagenesis and hybridization. For transgenic lines, describe the transformation method, the number of independent lines analyzed and the generation upon which experiments were performed. For gene-edited lines, describe the editor used, the endogenous sequence targeted for editing, the targeting guide RNA sequence (if applicable) and how the editor was applied.*
Authentication	*Describe any authentication procedures for each seed stock used or novel genotype generated. Describe any experiments used to assess the effect of a mutation and, where applicable, how potential secondary effects (e.g. second site T-DNA insertions, mosiacism, off-target gene editing) were examined.*
