# Supplementary material for: VPS13C/PARK23 initiates lipid transfer and membrane remodeling for efficient lysosomal repair
Source: Nat Commun. 2026 Jul 2;17:5789. doi: 10.1038/s41467-026-75145-y (PMC13328759; doi:10.1038/s41467-026-75145-y)
Supplement: Supplementary file 7 — Transparent Peer Review file [file 41467_2026_75145_MOESM7_ESM.pdf]

nature portfolio

# Peer Review File

# VPS13C/PARK23 initiates lipid transfer and membrane remodeling for efficient lysosomal repair

Corresponding Author: Professor Joost Holthuis

**This manuscript has been previously reviewed at another journal. This document only contains information relating to versions considered at Nature Communications. Mentions of the other journal have been redacted.**

**This file contains all reviewer reports in order by version, followed by all author rebuttals in order by version.**

Version 0:

Reviewer comments:

Reviewer #2

(Remarks to the Author)
The authors addressed my concerns and comments, I recommend publication after transfer to this new journal.
The authors might want to consider my concern about calling 3 micron beads containing compartments lysosomes. The paper they cite as an argument for non-canonical endocytosis of 3 um beads in HeLa cells is really weak. The size of the particle to ingest by cells is textbook data about phagocytosis from many years of research/papers and they have decided to use one single paper that is not very strong to justify this.The fact that you have large organelles (hypotonic vacuolation) is not really the same. And, phagolysosomes are not the same as lysosomes. In any case, it is a shame that this nice study will lose credibility with cell biologists working on phagocytosis.

Reviewer #3

(Remarks to the Author)
In this revision of a a previous manuscript submitted to [REDACTED], the authors have carrefully addressed my points by additional experiments, clarifications, and textual changes. The use of a flipper probe strongly improves the manuscript. The chemical biology approach to follow lipid flows is very informative. Irecommend publication

Reviewer #5

(Remarks to the Author)
In this revised manuscript, the authors describe VPS13C lipid transporter as a key regulator of the repair of damaged lysosomes that mediates the required bulk transfer of lipids to the lysosome upstream of PI4P-regulated lipid transport by OSPB and ORP lipid transporters.

In his/her review, referee #1 mainly criticizes the lack of novelty because VPS13C was previously described as an important lipid transporter in the repair of damaged lysosomes by the De Camilli lab.
This referee agrees with the authors that the new manuscript by the Holthuis lab provides key additional aspects and clarifies critical questions in addition to the previous study, which justify publication. Most prominently, the manuscript shows that VPS13C in fact is upstream of other lipid transfer events and that it mediates the critical net lipid transport from the ER to the lysosome (whereas other events exchange lipids). This key point is demonstrated by an elegant and unprecedented in-cell assay for lipid transfer, which represents a major advancement. The authors further conclusively show that factors (namely OSBP and ORPs) depend on prior function of VPS13C. Lastly, they demonstrate that VPS13C is recruited to "stressed" cells (before they get permeabilized) through RAB7-mediated binding, showing that RAB7-mediated and the damage-induced ATG2C-mediated recruitment can be temporally uncoupled. In contrast, the previous paper reported a coupled mechanism.

Assessment of the individual points and how they are addressed:

(1) The referee points out that differences in VCP13C recruitment to microbead-loaded versus dextran-loaded organelles could be due to timing differences and endocytic perturbation.
While the models may represent slightly different organelles (phagolysosomes versus endolysosomes), also phagolysosomes (for example containing bacteria) are established and relevant models for the damage response. In support of that, the authors recapitulate the point of tension-subjected membranes recruiting VPS13C by comparing the bead-filled organelles with hypoosmotic swelling of lysosomes, which sufficiently addresses this point.
(2) The referee suggests that VPS13C may activate OSBP by recruiting PI4K2A on ATG9 vesicles. The authors clarify that VPS13C does not help mobilize PI4K2A, which is also consistent with the previous paper showing no link of VPS13C to PI4P.
(3) The referee raised questions about how VPS13C-KO or PI4K2A-KO affect Apilimod-mediated lysosome swelling and LLOMe-induced damage which is clarified by the authors.
(4 & 7) The referee criticizes lack of citations on how ESCRT-mediated repair is supported by upstream signaling. The authors rightly argue that this is not the focus of the current study.
(5) The referee asks for clarification whether lysosomal membrane repair or invagination can occur independent of lipid transfer. The authors address this point by referring to data in the revised manuscript showing that lipid transfer is needed for repair, supporting the relevance of their finding. They further refer to the text that discusses how repair would be mediated by membrane invagination supported by lipid transfer.
(6) The referee asks for clarification on the mechanisms of VPS13C recruitment in hypotonic conditions (stressed state) versus LLOMe-mediated damage, also asking for definition of the stress sensor domain.
The authors refer to the requirement of the VAB domain that binds RAB7 in their experiments partially refuting this point. However, the nature of the "stressed membrane state" and the downstream signal from the membrane that recruits VPS13C (through activation of Rab7) admittedly still remains unclear.
(7) see point (4).
(8) The explanation why the pH sensor is not recovered in persisting LLOMe treatments is convincing. This condition does not affect the conclusions in the respective experiments.
(9) The concerns of the referee with respect to "membrane remodeling" are justified and poorly addressed by the authors. The term is ill-defined and only mention twice in the manuscript (title and model figure legends). To this referee, it remains unclear whether it is meant to describe OSBP/ORP recruitment, VAPA clustering or ER wrapping. The authors need to explain it better and use the term when describing the respective observations.

Conclusion:
In summary, most points of the referee have been addressed or refuted. A possible remaining weakness identified by referee #1 is that the nature of the proposed "stressed" membrane state and how this state signals to VPS13C recruitment (albeit through RAB7) remains undefined. In addition, the term "membrane remodeling" should be defined and used better.

**Open Access** This Peer Review File is licensed under a Creative Commons Attribution 4.0 International License, which permits use, sharing, adaptation, distribution and reproduction in any medium or format, as long as you give appropriate credit to the original author(s) and the source, provide a link to the Creative Commons license, and indicate if changes were made.
In cases where reviewers are anonymous, credit should be given to 'Anonymous Referee' and the source.
The images or other third party material in this Peer Review File are included in the article’s Creative Commons license,

unless indicated otherwise in a credit line to the material. If material is not included in the article's Creative Commons license and your intended use is not permitted by statutory regulation or exceeds the permitted use, you will need to obtain permission directly from the copyright holder.
To view a copy of this license, visit https://creativecommons.org/licenses/by/4.0/

**Response to reviewers' comments**

For clarity, comments from the reviewers are displayed in blue followed by our responses in black. We thank the reviewers for their time and the positive feedback on the revised manuscript.

**Reviewer #2**

The authors addressed my concerns and comments, I recommend publication after transfer to this new journal. The authors might want to consider my concern about calling 3 micron beads containing compartments lysosomes. The paper they cite as an argument for non-canonical endocytosis of 3 um beads in HeLa cells is really weak. The size of the particle to ingest by cells is textbook data about phagocytosis from many years of research/papers and they have decided to use one single paper that is not very strong to justify this. The fact that you have large organelles (hypotonic vacuolation) is not really the same. And, phagolysosomes are not the same as lysosomes. In any case, it is a shame that this nice study will lose credibility with cell biologists working on phagocytosis.

We thank the reviewer for the positive assessment of the revised manuscript. We acknowledge the reviewer's remaining concern and did not intent to call into question many years of research on the phagocytic uptake of particles, even though the number of papers focusing on non-professional phagocytosis of microbeads is limited. As shown in Figure 4b and Supplementary Figure 4a, microbeads functionalized with the pH-sensitive dye pHrodo began to fluoresce and localized within LAMP1-positive membranes shortly after cellular uptake, suggesting delivery to a lysosomal compartment. This led us to refer to these compartments as phagolysosomes or microbead-containing lysosomes (see p. 7, lines 228-232).

**Reviewer #3**

In this revision of a a previous manuscript submitted to [REDACTED], the authors have carrefully addressed my points by additional experiments, clarifications, and textual changes. The use of a flipper probe strongly improves the manuscript. The chemical biology approach to follow lipid flows is very informative. Irecommend publication.

We thank the reviewer for the positive assessment of the revised manuscript.

**Reviewer #5**

In this revised manuscript, the authors describe VPS13C lipid transporter as a key regulator of the repair of damaged lysosomes that mediates the required bulk transfer of lipids to the lysosome upstream of PI4P-regulated lipid transport by OSPB and ORP lipid transporters.

In his/her review, referee #1 mainly criticizes the lack of novelty because VPS13C was previously described as an important lipid transporter in the repair of damaged lysosomes by the De Camilli lab.
This referee agrees with the authors that the new manuscript by the Holthuis lab provides key additional aspects and clarifies critical questions in addition to the previous study, which justify publication. Most prominently, the manuscript shows that VPS13C in fact is upstream of other lipid transfer events and that it mediates the critical net lipid transport from the ER to the lysosome (whereas other events exchange lipids). This key point is demonstrated by an elegant and unprecedented in-cell assay for lipid transfer, which represents a major advancement. The

authors further conclusively show that factors (namely OSBP and ORPs) depend on prior function of VPS13C. Lastly, they demonstrate that VPS13C is recruited to "stressed" cells (before they get permeabilized) through RAB7-mediated binding, showing that RAB7-mediated and the damage-induced ATG2C-mediated recruitment can be temporally uncoupled. In contrast, the previous paper reported a coupled mechanism.

Assessment of the individual points and how they are addressed:

(1) The referee points out that differences in VCP13C recruitment to microbead-loaded versus dextran-loaded organelles could be due to timing differences and endocytic perturbation. While the models may represent slightly different organelles (phagolysosomes versus endolysosomes), also phagolysosomes (for example containing bacteria) are established and relevant models for the damage response. In support of that, the authors recapitulate the point of tension-subjected membranes recruiting VPS13C by comparing the bead-filled organelles with hypoosmotic swelling of lysosomes, which sufficiently addresses this point.
(2) The referee suggests that VPS13C may activate OSBP by recruiting PI4K2A on ATG9 vesicles. The authors clarify that VPS13C does not help mobilize PI4K2A, which is also consistent with the previous paper showing no link of VPS13C to PI4P.
(3) The referee raised questions about how VPS13C-KO or PI4K2A-KO affect Apilimod-mediated lysosome swelling and LLOMe-induced damage which is clarified by the authors.
(4 & 7) The referee criticizes lack of citations on how ESCRT-mediated repair is supported by upstream signaling. The authors rightly argue that this is not the focus of the current study.
(5) The referee asks for clarification whether lysosomal membrane repair or invagination can occur independent of lipid transfer. The authors address this point by referring to data in the revised manuscript showing that lipid transfer is needed for repair, supporting the relevance of their finding. They further refer to the text that discusses how repair would be mediated by membrane invagination supported by lipid transfer.
(6) The referee asks for clarification on the mechanisms of VPS13C recruitment in hypotonic conditions (stressed state) versus LLOMe-mediated damage, also asking for definition of the stress sensor domain.
The authors refer to the requirement of the VAB domain that binds RAB7 in their experiments partially refuting this point. However, the nature of the "stressed membrane state" and the downstream signal from the membrane that recruits VPS13C (through activation of Rab7) admittedly still remains unclear.
(7) see point (4).
(8) The explanation why the pH sensor is not recovered in persisting LLOMe treatments is convincing. This condition does not affect the conclusions in the respective experiments.
(9) The concerns of the referee with respect to "membrane remodeling" are justified and poorly addressed by the authors. The term is ill-defined and only mention twice in the manuscript (title and model figure legends). To this referee, it remains unclear whether it is meant to describe OSBP/ORP recruitment, VAPA clustering or ER wrapping. The authors need to explain it better and use the term when describing the respective observations.

Conclusion:
In summary, most points of the referee have been addressed or refuted. A possible remaining weakness identified by referee #1 is that the nature of the proposed "stressed" membrane state and how this state signals to VPS13C recruitment (albeit through RAB7) remains undefined. In addition, the term "membrane remodeling" should be defined and used better.

We thank the reviewer for the comprehensive evaluation of our response to the original criticisms raised by Reviewer #1 and for the positive assessment of the revised manuscript.

As stated in the manuscript, we acknowledge that elucidation of the mechanism by which VPS13C gains access to lysosome-bound Rab7 in response to lysosomal stress will require more work (see p. 9, lines 300-303; p. 14, lines 519-528).

To clear up any confusion regarding the use of the term “membrane remodeling”, we have added a new reference in which this process is described (Subra et al., Ref. 22) and modified the Introduction and Discussion as indicated below (textual changes marked in red).

**Introduction** (p. 4, lines 97-100)
Since OSBP and ORPs selectively transport cholesterol and phosphatidylserine (PS) from the ER to the lysosome-limiting membrane in exchange for PI4P, they do not mediate net lipid transfer to damaged lysosomes but primarily serve a role in membrane remodeling[22].

**Discussion** (p. 13, lines 527-531)
… OSBP and ORPs subsequently catalyse ER-to-lysosome transfer of cholesterol and PS in exchange for lysosome-derived PI4P, thereby enabling a remodeling of the lysosome-limiting membrane. Although this counter lipid transport does not result in net lipid transfer to lysosomes, the ORP-mediated accumulation of PS on the lysosomal surface stimulates the delivery of bulk lipids for lysosomal repair by the bridge-like lipid transport protein ATG2A.
